# Thromboxane biosynthesis in cancer patients and its inhibition by aspirin: a sub-study of the Add-Aspirin trial

**DOI:** 10.1038/s41416-023-02310-1

**Published:** 2023-07-07

**Authors:** Nalinie Joharatnam-Hogan, Duaa Hatem, Fay H. Cafferty, Giovanna Petrucci, David A. Cameron, Alistair Ring, Howard G. Kynaston, Duncan C. Gilbert, Richard H. Wilson, Richard A. Hubner, Daniel E. B. Swinson, Siobhan Cleary, Alex Robbins, Mairead MacKenzie, Martin W. G. Scott-Brown, Sharmila Sothi, Lesley K. Dawson, Lisa M. Capaldi, Mark Churn, David Cunningham, Vincent Khoo, Anne C. Armstrong, Nicola L. Ainsworth, Gail Horan, Duncan A. Wheatley, Russell Mullen, Fiona J. Lofts, Axel Walther, Rebecca A. Herbertson, John D. Eaton, Ann O’Callaghan, Andrew Eichholz, Mohammed M. Kagzi, Daniel M. Patterson, Krishna Narahari, Jennifer Bradbury, Zuzana Stokes, Azhar J. Rizvi, Georgina A. Walker, Victoria L. Kunene, Narayanan Srihari, Aleksandra Gentry-Maharaj, Angela Meade, Carlo Patrono, Bianca Rocca, Ruth E. Langley

**Affiliations:** 1 MRC Clinical Trials Unit, UCL, London, UK; 2 Department of Safety and Bioethics, Division of Pharmacology, Catholic University School of Medicine, Rome, Italy; 3 The Institute of Cancer Research, London, UK; 4 Cancer Research UK Edinburgh Centre, Institute of Genetics and Cancer, The University of Edinburgh, Western General Hospital, Edinburgh, UK; 5 The Royal Marsden NHS Foundation Trust, London, UK; 6 Department of Urology, Cardiff University School of Medicine, Cardiff, UK; 7 School of Cancer Sciences, University of Glasgow, Glasgow, UK; 8 The Beatson West of Scotland Cancer Centre, Glasgow, UK; 9 The Christie NHS Foundation Trust, Department of Medical Oncology, Manchester, UK; 10 University of Manchester, Division of Cancer Sciences, Manchester, UK; 11 Leeds Teaching Hospitals NHS Trust, Leeds, UK; 12 Independent Cancer Patients’ Voice, London, UK; 13 University Hospitals Coventry and Warwickshire NHS Trust, Coventry, UK; 14 Edinburgh Cancer Centre, Western General Hospital, Edinburgh, UK; 15 Worcestershire Acute Hospitals NHS Trust, Worcester, UK; 16 The Queen Elizabeth Hospital King’s Lynn NHS Foundation Trust, King’s Lynn, UK; 17 Royal Cornwall Hospitals NHS Trust, Cornwall, UK; 18 The Highland Breast Centre, Raigmore Hospital, Inverness, UK; 19 St George’s University Hospitals NHS Foundation Trust, London, UK; 20 University Hospitals Bristol and Weston NHS Foundation Trust, Bristol, UK; 21 University Hospitals Sussex NHS Foundation Trust, Brighton, UK; 22 University Hospitals of Morecambe Bay NHS Foundation Trust, Kendal, UK; 23 Portsmouth Hospitals University NHS Trust, Portsmouth, UK; 24 Buckinghamshire Healthcare NHS Trust, Buckinghamshire, UK; 25 South Tees Hospitals NHS Foundation Trust, Middlesbrough, UK; 26 West Suffolk Hospitals NHS Trust, St Edmunds, UK; 27 University Hospital of Wales, Cardiff and Vale University Health Board, Cardiff, UK; 28 Division of Cancer and Genetics, Cardiff University, Cardiff, UK; 29 Salisbury NHS Foundation Trust, Salisbury, UK; 30 United Lincolnshire Hospitals NHS Trust, Lincoln City, UK; 31 Milton Keynes University Hospital NHS Foundation Trust, Milton Keynes, UK; 32 Nottingham University Hospitals NHS Trust, Nottingham, UK; 33 Walsall Manor Hospital and University Hospitals, Birmingham NHS Foundation Trust, Birmingham, UK; 34 Shrewsbury and Telford Hospital NHS Trust, Shrewsbury, UK; 35 Present Address: University Hospitals Sussex NHS Foundation Trust, Brighton, UK

**Keywords:** Cancer, Cancer

## Abstract

**Background:**

Pre-clinical models demonstrate that platelet activation is involved in the spread of malignancy. Ongoing clinical trials are assessing whether aspirin, which inhibits platelet activation, can prevent or delay metastases.

**Methods:**

Urinary 11-dehydro-thromboxane B_2_ (U-TXM), a biomarker of in vivo platelet activation, was measured after radical cancer therapy and correlated with patient demographics, tumour type, recent treatment, and aspirin use (100 mg, 300 mg or placebo daily) using multivariable linear regression models with log-transformed values.

**Results:**

In total, 716 patients (breast 260, colorectal 192, gastro-oesophageal 53, prostate 211) median age 61 years, 50% male were studied. Baseline median U-TXM were breast 782; colorectal 1060; gastro-oesophageal 1675 and prostate 826 pg/mg creatinine; higher than healthy individuals (~500 pg/mg creatinine). Higher levels were associated with raised body mass index, inflammatory markers, and in the colorectal and gastro-oesophageal participants compared to breast participants (*P* < 0.001) independent of other baseline characteristics. Aspirin 100 mg daily decreased U-TXM similarly across all tumour types (median reductions: 77–82%). Aspirin 300 mg daily provided no additional suppression of U-TXM compared with 100 mg.

**Conclusions:**

Persistently increased thromboxane biosynthesis was detected after radical cancer therapy, particularly in colorectal and gastro-oesophageal patients. Thromboxane biosynthesis should be explored further as a biomarker of active malignancy and may identify patients likely to benefit from aspirin.

## Introduction

Activated platelets play a central role in the development and spread of cancer, as well as in haemostasis and thrombosis, tissue repair, and inflammation [1–3]. The association of platelets with malignancy was highlighted by Trousseau in 1865 with the observation that thrombosis could be an early sign of occult malignancy [4]. Pre-clinical experimental models demonstrate the interaction between platelets, circulating tumour cells, the tumour microenvironment and the development of metastases (reviewed in refs. [5–7]). Activated platelets are thought to protect circulating tumour cells from immune clearance, promote adhesion to the endothelium, facilitate transendothelial migration and the formation of metastatic niches.

The primary effect of once-daily low-dose (75–100 mg) aspirin is the irreversible acetylation of cyclo-oxygenase-1 (COX-1) in platelets [8, 9]. Thromboxane A_2_ (TXA_2_) is the main product of platelet arachidonic acid metabolism via COX-1, and amplifies platelet activation and aggregation in response to various stimuli [10]. Inhibition of COX-1 in platelets with aspirin, or abolition of TXA_2_ signalling by either genetic or pharmacological manipulation, in pre-clinical models, leads to a reduction in metastases attributed to an effect on circulating tumour cells and their interaction with platelets during intravascular transition [11, 12].

The link between TXA_2_-dependent platelet activation and increased COX-2 expression which is associated with carcinogenesis [13] has been strengthened by recent data obtained from genetically modified mice. Mice with a specific deletion of COX-1 in megakaryocytes/platelets (pPtgs1^−/−^) were crossed with Apc^min/+^ mice which develop multiple intestinal polyps and are considered a relevant model for studying intestinal carcinogenesis. The pPtgs1^−/−^/Apc^min/+^ mice with largely suppressed TXA_2_ biosynthesis developed significantly fewer intestinal adenomas than Apc^min/+^ mice, and the adenomas that did develop had a lower proliferative index and reduced COX-2 expression. This data suggests that the role of COX-1 in platelets in terms of promoting intestinal carcinogenesis could be mediated through the release of pro-inflammatory and pro-angiogenic autacoids to enhance aberrant adenomatous COX-2 expression [14]. Data have also shown an association between overexpression of TXA_2_ synthase and TXA_2_ receptors in multiple cancer types [15], as well as an unexpected association between a single nucleotide polymorphism in TBXA_2_ receptor, causing a gain in function, and a multiple metastatic phenotype across tumour types [16].

Epidemiological and randomised data demonstrate that once-daily aspirin use reduces cancer incidence particularly for tumours that arise from the gastrointestinal tract [17–20], and when individuals are at increased risk of developing malignancy. For example, aspirin has recently been recommended by the UK National Institute for Clinical Excellence (NICE) for the prevention of colorectal cancer in Lynch Syndrome (hereditary mismatch repair deficiency (MMRd)) [21] as a result of long-term data from the CAPP2 trial (ISCRTN59521990) [22]. Beyond this however, there is still uncertainty as to how aspirin can be best employed in the anti-cancer armamentarium. For primary prevention in the wider population, there are concerns about potential benefits being outweighed by the bleeding risk from aspirin [23, 24]. For the treatment of cancer/prevention of metastases, there are several randomised trials ongoing internationally to assess whether aspirin use after initial radical treatment, particularly for colorectal cancer, can prevent or delay the subsequent formation of metastases [25].

It is estimated that about 5% of patients who present with unexplained venous thromboembolism (VTE) develop cancer within 1 year of diagnosis. The risk of developing a cancer-associated VTE depends on the type and stage of the cancer, treatment received, as well as the underlying risk of the patient [26]. Several biomarkers of haemostasis for example d-dimer appear to be associated with VTE and cancer progression [27]. Clinical markers of platelet activation (for example soluble P-selectin) have also been shown to be associated with the development of thromboses in cancer patients [28]. In a cohort of 687 cancer patients (35% newly diagnosed with localised disease) the probability of a VTE during the first 6 months of the study was 11.9% for those with a serum P-selectin above the 75th percentile and 3.7% for those below [29]. However, in this study other markers of platelet activation (soluble CD40 ligand, thrombospondin-1 (TSP-1) and platelet factor-4 (PF-4)) were not associated with VTE, and haemostatic biomarkers are not routinely measured in oncological practice.

TXA_2_ is chemically unstable in aqueous solution with a very short half-life ~30 s [30]. It hydrolyses non-enzymatically to the inactive, but chemically stable, urinary 11-dehydro-thromboxane B_2_ (U-TXM) a major enzymatic metabolite of TXA_2_/TXB_2_ [31]. U-TXM provides a non-invasive index or biomarker of in vivo TXA_2_ biosynthesis. U-TXM has been used extensively to characterise clinical scenarios associated with enhanced platelet activation within the fields of cardiology and onco-haematology, and to guide the design of low-dose aspirin trials in these settings [31–33]. More recently, high excretion rates of U-TXM in a cohort of the Framingham Heart Study (*n* = 3044) have been associated with increased cancer mortality (hazard ratio (HR) 1.63, 95% confidence interval (CI) 1.24–2.13, *P* = 0.0005), as well as cardiovascular mortality (HR 2.41, 95% CI 1.71–3.39, *P* < 0.0001) and all-cause mortality (HR 1.96, 95% CI 1.68–2.29, *P* < 0.0001) [34].

Further understanding of the mechanism(s) underlying the anti-cancer effects of aspirin would permit a more nuanced and precision-medicine approach to the use of aspirin in the field of oncology. The ongoing adjuvant Add-Aspirin trial (ISRCTN74358648) which is evaluating the effect of aspirin use after radical treatment in four different tumour types and evaluating 2 different doses of aspirin, provided an opportunity to further study thromboxane biosynthesis in cancer patients at an early stage of their disease, and the short-term effects of two doses aspirin. The specific aims of this study were: (i) to examine the levels of thromboxane biosynthesis using U-TXM as a biomarker in cancer patients who had recently completed radical treatment and had no radiological or standard clinical evidence of metastatic disease, (ii) to evaluate the effects of aspirin 100 and 300 mg daily on U-TXM in this cohort and (iii) to provide information about participant treatment compliance in this blinded placebo-controlled trial particularly as aspirin is also available as an over-the-counter preparation.

## Methods

### Trial design and participants

This thromboxane biosynthesis study was embedded within the randomised Add-Aspirin trial framework. The Add-Aspirin protocol encompasses four individually powered, Phase III, adjuvant studies in four different tumour types, i.e., breast, colorectal, gastro-oesophageal and prostate. Full details of the trial have been published previously [35]. At trial entry, all participants start an open-label 8-week run-in phase of aspirin 100 mg daily to assess tolerability followed by random allocation to aspirin 100 mg, aspirin 300 mg or matched placebo for up to 5 years. Participants ≥75 years are only allocated to aspirin 100 mg or placebo. The timing of entry into the trial is tumour cohort-specific and dependent upon the radical treatment received. For those that had only received surgery the run-in phase could be started 6–12 weeks after surgery (and up to 16 weeks post-surgery for gastro-oesophageal cancer participants). Adjuvant chemotherapy could be ongoing at trial entry in the colorectal and gastro-oesophageal cohorts, and ongoing long-term hormonal treatment is permitted in the breast and prostate cohorts. The main exclusion criteria include regular aspirin use within the previous 5 years and concomitant medication/previous medical history that would increase the risk of aspirin toxicity.

All participants (*n* = 716) in this sub-study had histologically confirmed adenocarcinoma (except four participants had squamous cell histology and one adenosquamous in the gastro-oesophageal cohort). At study entry, all participants had no previous or current evidence of metastatic disease with the exception of four patients in the colorectal cohort who had completely resected liver metastases. The specific eligibility criteria for each tumour cohort were designed to identify patients who had received treatment with radical intent but were at moderate to high risk of relapse. For breast participants who had not received neoadjuvant chemotherapy the final histology had to include lymph node positivity or, if node-negative, have at least two high-risk features (defined as: oestrogen receptor (ER) negative, human epidermal growth factor receptor 2 (HER2) positive, grade 3, lymphovascular invasion present, age <35 years, oncotype dx score >25 or prosignia score prediction analysis of microarray 50 (PAM50) > 60). Participants who received neoadjuvant chemotherapy or radiotherapy should not have achieved a complete pathological response and be either (i) HER2 positive, (ii) HER2 negative and grade 3 or (iii) HER2 negative and ER-negative. Colorectal cancer participants were eligible if, following a surgical resection, they had a pathological Stage II (T3-4 N0) or Stage III (T1-4 N1 or N2) tumour. For those that received neoadjuvant therapy (*n* = 8), staging is based on pre-treatment assessments. Gastro-oesophageal participants had to have undergone surgery with radical intent with a post-operative pathological stage of T1b-T4, N any, M0, or received radical radiotherapy. Prostate cancer participants were node-negative with a clinical or radiological Stage 1-3b and D’Amico classification intermediate risk (at least 1 of prostate-specific antigen [PSA] between 10 and 20, Gleason score of 7 or clinical stage T2b) or high risk (PSA >20, or Gleason score >8 or clinical stage T2c-3a).

The trial protocol, including the thromboxane biosynthesis study, was approved by South Central-Oxford C research ethics committee (Ref: 14/SC/0171) and by local research and development departments at all centres. Participants provided written informed consent.

### U-TXM study

**Fig. 1:**
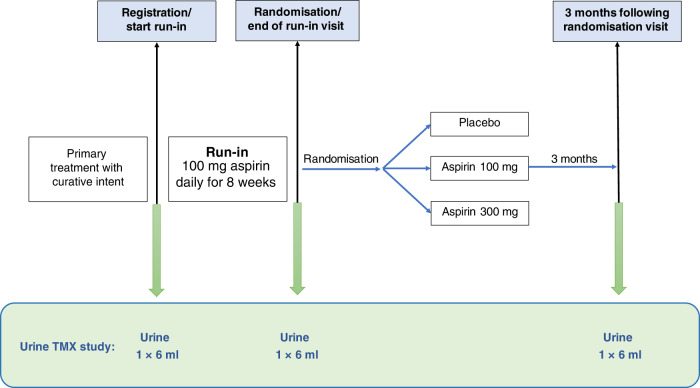
U-TXM study. Urine samples were collected at trial entry (off aspirin), after an 8-week run-in period of aspirin 100 mg daily for all participants and 3–6 months after random allocation to aspirin 100 mg, aspirin 300 mg or matched placebo daily.

The thromboxane biosynthesis study was undertaken at 28 UK centres. Participants provided 6-ml urine samples at trial entry/baseline (off aspirin), after the 8-week run-in period of aspirin 100 mg daily (24 h after the previous aspirin dose), and a third sample between 3 and 6 months after randomisation to blinded treatment (aspirin 100 mg, aspirin 300 mg or matched placebo) (Fig. 1) using a minimisation algorithm with a random element.

End of run-in, and post-randomisation, urine samples were only included in this analysis if the participant had remained on allocated treatment. Adherence was assessed by participant self-reporting and review of used blister packs/completion of diary cards at the end of the run-in period. Based on these assessments participants were included if they had taken 6–7 tablets per week and had not discontinued treatment prior to the urine sample being obtained.

Urine samples were sent by post from trial centres to one of two trial biobanks in the UK within 24 h of collection. Aliquoted into 2-ml cryo-vials, frozen at −20 °C or below and transferred on dry ice to the Institute of Pharmacology at the Catholic University School of Medicine (Rome, Italy). The extraction procedure was performed on 1 ml urine samples as described in detail in Supplementary Appendix S1 and [36]. U-TXM was measured with a standard Enzyme-Linked Immunosorbent Assay (ELISA) [36–38]; 96-well plates were coated with commercial monoclonal anti-rabbit IgG antibodies (Cayman Chemical, Ann Arbor, MI, USA) and U-TXM assessed with a standard acetylcholinesterase ELISA immunometric method using a specific rabbit polyclonal antibody. The cross-reactivity of the anti-11-dehydro-TXB_2_ antibody against other prostanoids that can be measured in urine, namely PGE_2,_ TXB_2_, 6-keto PGF_1alpha_, and the isoprostane 8-iso-PGF_2alpha_ was <0.05% [39]. We also tested cross-reactivity with 2,3-dinor-TXB2 which was <1.5% (data not shown). Results were expressed per milligram of urinary creatinine measured with a commercial kit (Creatinine Colorimetric Detection Kit; Enzo Life Sciences, Farmingdale, NY, USA). The authors involved in U-TXM measurements (DH, GP and BR) were blinded to all clinical details and aspirin/placebo allocation.

### Statistical analysis

For the breast, colorectal and prostate cohorts with a sample size of ~200 for each cohort the study was powered to estimate the baseline mean U-TXM with a precision of +/− 40 pg/mg creatinine with a 95% CI based on the U-TXM distribution of healthy individuals (mean 504 pg/mg, standard deviation 267 pg/mg) [31]. For the gastro-oesophageal cohort where the sample size was smaller (*n* = 53) the expected precision was +/− 75 pg/mg based on a 95% CI. The primary outcomes of the analysis were to (i) estimate thromboxane biosynthesis following radical cancer therapy (at baseline); and (ii) establish the effects of daily aspirin at 100 mg and 300 mg on this parameter.

Correlations between U-TXM levels and baseline subject demographics including age, male/female, weight as body mass index (BMI), tumour type, comorbidity, smoking status, concurrent medication, recent/ongoing cancer treatment, risk of recurrence, baseline blood counts including platelet number and inflammatory markers were explored. A higher risk of recurrence was defined for each tumour cohort based on American Joint Committee on Cancer (AJCC) TNM staging (7th edition) as follows: breast Stage III (Tany N2, T3 N1-2, T4 Nany), colorectal Stage III (Tany N1-2) or Stage IV, gastro-oesophageal Stage IIB or III and for prostate cancer D’Amico high risk as previously defined. Summary statistics and plots explored associations, then linear regression models were fitted. Regression models were based on log-transformed values of U-TXM to optimise model fit. Univariable models were fitted initially, then a multivariable model was constructed using forward stepwise procedures considering all of the covariates listed (*P* value to enter a term <0.05; *P* value ≥ 0.1 to remove a term). Interactions were considered, though power was low for this.

Summary statistics were used to consider U-TXM values at the end of the run-in period (by tumour cohort) and after 3 months of randomised treatment (by treatment allocation). Participants ≥75 years were not included in the analysis of dose as they were not eligible for random allocation to the higher aspirin dose. End of run-in U-TXM (adjusted for baseline values) was considered according to baseline characteristics and previous therapy in univariable and multivariable linear models (based on log-transformed U-TXM values). U-TXM during randomised treatment is presented according to the treatment group and baseline factors, but modelling was not performed due to small numbers.

## Results

Between May 2018 and January 2021, 716 participants provided a urine sample at trial entry with the number of follow-up samples shown in the consort diagram (Supplementary Appendix S2). The number of participants in each tumour cohort (including the proportion not proceeding from run-in to randomisation (12% in this study and 15% in the trial overall), and the baseline characteristics (Table 1A) were in line with the trial more broadly [40]. The median age of the whole cohort was 61 years with the expected male: female ratio within each tumour type, 27% with a BMI ≥ 30 kg/m^2^, 42% previous and 5% current smokers. Details of the radical treatment received are also in Table 1A. Thirty-five percent had just received surgery (most common in the prostate cohort), 49% had undergone surgery followed by adjuvant chemotherapy and 14% radical radiotherapy or chemoradiation. Twenty-one percent were within 6–12 weeks of surgery and for only 17 (2%) was the adjuvant chemotherapy still ongoing.

**Table 1:** Participant demographics and baseline U-TXM data. Panel A: participant characteristics and panel B: U-TXM at trial registration cohort.

(A)					
	Breast	Colorectal	Gastro-oesophageal	Prostate	All
Number of participants	260	192	53	211	716
**Gender**					
Male	1, <1%	104, 54%	40, 75%	211, 100%	356, 50%
Female	259, >99%	88, 46%	13, 25%	0, 0%	360, 50%
**Age at registration (years)**					
Median (IQR)	53 (47–62)	61 (52–69)	63 (58–69)	67 (62–72)	61 (52–68)
Range	25–80	21–81	43–79	48–83	21–83
Under 50 years	88, 34%	34, 18%	6, 11%	1, <1%	129, 18%
50–59 years	94, 36%	53, 28%	13, 25%	32, 15%	192, 27%
60–69 years	58, 22%	59, 31%	21, 40%	108, 51%	246, 34%
70 years or older	20, 8%	46, 24%	13, 25%	70, 33%	149, 21%
**BMI**					
Median (Q1–Q3)	27 (24–32)	27 (24–31)	25 (23–29)	28 (25–31)	27 (24–31)
Range	18–45	19–47	16–33	20–45	16–47
Missing	21	13	7	11	52
Under 25	79, 30%	58, 30%	22, 42%	44, 21%	203, 28%
25–29.9	80, 31%	71, 37%	17, 32%	97, 46%	265, 37%
30–34.9	42, 16%	34, 18%	7, 13%	41, 19%	124, 17%
35 or more	38, 15%	16, 8%	0, 0%	18, 9%	72, 10%
Missing	21, 8%	13, 7%	7, 13%	11, 5%	52, 7%
**Smoking status**					
Never	154, 59%	93, 48%	19, 36%	109, 52%	375, 52%
Ex-smoker	94, 36%	88, 46%	30, 57%	91, 43%	303, 42%
Current smoker	12, 5%	11, 6%	4, 8%	11, 5%	38, 5%
**Diabetic**					
No	232, 89%	165, 86%	44, 83%	189, 90%	630, 88%
Yes	5, 2%	16, 8%	6, 11%	11, 5%	38, 5%
Missing	23, 9%	11, 6%	3, 6%	11, 5%	48, 7%
**Receiving BP treatmen**t					
No	200, 77%	135, 70%	33, 62%	124, 59%	492, 69%
Yes	38, 15%	44, 23%	17, 32%	74, 35%	173, 24%
Missing	22, 8%	13, 7%	3, 6%	13, 6%	51, 7%
**Statins taken within the last week**					
No	236, 91%	148, 77%	38, 72%	144, 68%	566, 79%
Yes	11, 4%	33, 17%	13, 25%	54, 26%	111, 16%
Missing	13, 5%	11, 6%	2, 4%	13, 6%	39, 5%
**Neutrophils (10**^**9**^**/L)**					
<5.0	227, 87%	159, 83%	43, 81%	172, 82%	601, 84%
≥5.0	20, 8%	25, 13%	8, 15%	29, 14%	82, 11%
Missing	13, 5%	8, 4%	2, 4%	10, 5%	33, 5%
**Total WCC (10**^**9**^**/L)**					
<8.0	237, 91%	162, 84%	42, 79%	184, 87%	625, 87%
≥8.0	9, 3%	22, 11%	9, 17%	16, 8%	56, 8%
Missing	14, 5%	8, 4%	2, 4%	11, 5%	35, 5%
**Platelet count (10**^**9**^**/L)**					
<350	230, 88%	173, 90%	44, 83%	196, 93%	643, 90%
≥350	13, 5%	12, 6%	7, 13%	5, 2%	37, 5%
Missing	17, 7%	7, 4%	2, 4%	10, 5%	36, 5%
**Raised LDL, total cholesterol or non-HDL**^a^					
No	70, 27%	81, 42%	31, 58%	88, 42%	270, 38%
Yes	154, 59%	92, 48%	18, 34%	98, 46%	362, 51%
Missing	36, 14%	19, 10%	4, 8%	25, 12%	84, 12%
**CRP result (categorised)**					
≤5	208, 80%	145, 76%	41, 77%	168, 80%	562, 78%
>5	28, 11%	30, 16%	8, 15%	23, 11%	89, 12%
Missing	24, 9%	17, 9%	4, 8%	20, 9%	65, 9%
**Higher-risk disease**^b^					
No	183, 70%	78, 41%	10, 19%	71, 34%	342, 48%
Yes	66, 25%	111, 58%	42, 79%	137, 65%	356, 50%
Missing	11, 4%	3, 2%	1, 2%	3, 1%	18, 3%
**Primary treatment details**					
Surgery, no adjuvant chemo	84, 32%	44, 23%	10, 19%	112, 53%	250, 35%
Surgery with adjuvant chemo	166, 64%	145, 76%	39, 74%	0, 0%	350, 49%
Radical RT or CRT	0, 0%	0, 0%	3, 6%	97, 46%	100, 14%
Missing details (surgery pts)	10, 4%	3, 2%	1, 2%	2, <1%	16, 2%
**Weeks between surgery and registration (n = 616)**^c^					
6–12 weeks	12, 5%	42, 22%	9, 17%	90, 43%	153, 21%
>12 weeks	233, 90%	143, 74%	40, 75%	20, 9%	436, 61%
Missing	15, 6%	7, 4%	1, 2%	4, 2%	27, 4%
**Timing of entry relative to adjuvant chemotherapy (n = 366)**					
After chemo has finished	166, 64%	129, 67%	38, 72%	0, 0%	333, 47%
While chemo is ongoing	0, 0%	16, 8%	1, 2%	0, 0%	17, 2%
Missing	10, 4%	3, 2%	1, 2%	2, <1%	16, 2%

(B)				
Baseline characteristic	*n*	Median	(Q1, Q3)	[Range]
**Tumour type**				
Breast	260	782	(503, 1178)	[114, 5999]
Colorectal	192	1060	(667, 1558)	[159, 5159]
Gastro-oesophageal	53	1675	(1150, 2365)	[415, 5182]
Prostate	211	826	(555, 1173)	[98, 3866]
**Gender**				
Male	356	966	(636, 1431)	[98, 4957]
Female	360	839	(530, 1314)	[114, 5999]
**Age at registration (years)**				
Under 50 years	129	778	(533, 1294)	[141, 4383]
50–59 years	192	911	(572, 1342)	[114, 5999]
60–69 years	246	867	(565, 1315)	[98, 4957]
70 years or older	149	1001	(652, 1509)	[112, 5182]
**BMI**				
BMI < 25	203	904	(590, 1385)	[103, 4957]
BMI 25–29.9	265	886	(609, 1337)	[112, 5182]
BMI 30–34.9	124	813	(528, 1352)	[98, 3526]
BMI > 35	72	1011	(640, 1543)	[225, 5999]
**Smoking status**				
Never	375	834	(526, 1248)	[103, 5999]
Ex-smoker	303	976	(602, 1477)	[98, 5182]
Current smoker	38	1379	(750, 2182)	[466, 4957]
**Diabetic**				
Not diabetic	630	894	(575, 1361)	[112, 5182]
Diabetic	38	986	(745, 1531)	[98, 5999]
**Receiving BP treatment**				
No BP treatment	492	909	(591, 1347)	[103, 5999]
On BP treatment	173	851	(569, 1387)	[98, 4714]
**Statin use in last week**				
No statins	566	908	(597, 1366)	[103, 5182]
Statin use (within last week)	111	859	(500, 1379)	[98, 5999]
**Neutrophils (10**^**9**^**/L)**				
Neutrophils <5.0 × 10^9^/L	601	879	(559, 1330)	[98, 5159]
Neutrophils ≥5.0 × 10^9^/L	82	1134	(735, 1761)	[239, 5999]
**Total WCC (10**^**9**^**/L)**				
Total WCC < 8.0 × 10^9^/L	625	886	(566, 1330)	[98, 5159]
Total WCC ≥ 8.0 × 10^9^/L	56	1212	(817, 2089)	[426, 5999]
**Platelet count (10**^**9**^**/L)**				
Platelet count <350 × 10^9^/L	643	887	(569, 1343)	[98, 5999]
Platelet count ≥350 × 10^9^/L	37	1244	(834, 1777)	[185, 4957]
**Raised LDL, total cholesterol or non-HDL**				
Normal cholesterol	270	920	(584, 1403)	[98, 5999]
Raised LDL, total cholesterol or non-HDL^a^	362	898	(568, 1342)	[114, 5182]
**CRP result (categorised)**				
CRP ≤ 5	562	875	(573, 1354)	[98, 5999]
CRP > 5	89	1079	(690, 1755)	[159, 5182]
**Higher-risk disease**				
Lower-risk disease	342	842	(531, 1342)	[114, 4957]
Higher-risk disease^b^	356	968	(650, 1400)	[98, 5999]
**Primary treatment details**				
Surgery, no adjuvant chemo	250	828	(525, 1194)	[103, 4957]
Surgery with adjuvant chemo	350	1021	(653, 1558)	[114, 5999]
Radical RT or CRT	100	824	(543, 1183)	[98, 2948]
**Time between surgery and registration**				
<12 weeks since surgery	153	904	(597, 1231)	[103, 4957]
≥12 weeks since surgery	436	934	(599, 1484)	[114, 5999]
**Timing of entry relative to adjuvant chemotherapy**				
Surgery without adjuvant chemo	250	828	(525, 1194)	[103, 4957]
Surgery and adj chemo—completed	333	986	(613, 1531)	[114, 5999]
Surgery and adj chemo—ongoing	17	1465	(1283, 1678)	[749, 2769]
All patients	716	898	(572, 1366)	[98, 5999]

(B) U-TXM at registration according to baseline factors.

^a^LDL > 3, total cholesterol>5 or non-HDL > 4 defined as elevated; note that participants with at least one of LDL, HDL and total cholesterol measured and in the normal range (and no raised measurements) are included in the normal group even if other measurements are missing.

^b^High-risk disease defined as breast Stage III; colorectal Stage III or IV; gastro-oesophageal Stage IIB or III; prostate high risk according to D’Amico classification.

^c^Participants could not register for the trial until at least 6 weeks post-surgery.

Neutrophil, platelet and total white cell categories based on upper quartile and above.

### Baseline levels of platelet activation after radical treatment for early-stage cancers

**Fig. 2:**
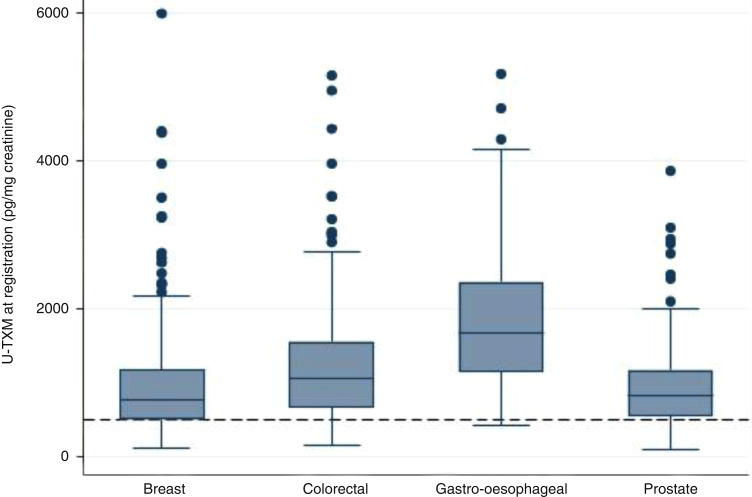
Distribution of U-TXM at registration by tumour-specific cohort—breast (*n* = 260), colorectal (*n* = 192), gastro-oesophageal (*n* = 53), prostate (*n* = 211). The dotted line represents the median value in healthy individuals [31].

Median U-TXM excretion was higher across all the tumour cohorts than observed previously in studies of healthy individuals where the median values were around 500 pg/mg creatinine (Fig. 2, Table 1B and Supplementary Appendix S3) [31, 41, 42]. Median values (quartile 1, quartile 3) for each cohort were: breast 782 pg/mg (503, 1178), colorectal 1060 pg/mg (667, 1558), gastro-oesophageal 1675 pg/mg (1150, 2365) and prostate 826 pg/mg (555, 1173). The individual data is shown in Supplementary Appendix S4. Higher median values (significantly >1000 pg/mg) were associated with the gastro-oesophageal cohort, current smoking, raised neutrophils, total white cells and platelet counts, as well as ongoing adjuvant chemotherapy. Similar levels of U-TXM were seen in participants who were 6–12 weeks from surgery compared to those that were ≥12 weeks, as well in those that had surgery (with no adjuvant chemotherapy) compared to radical radiotherapy or chemoradiotherapy (Table 1B and Appendix S5). There was also no significant difference in U-TXM levels between those considered to be at the highest risk of relapse (as defined above) compared to the whole cohort. In multivariable analyses, higher U-TXM excretion was independently associated with tumour type, specifically the colorectal and gastro-oesophageal cohorts compared to the breast cohort (both *P* < 0.001), BMI ≥ 35 kg/m^2^ (*P* = 0.008), neutrophils ≥5.0 × 10^9^/l (*P* = 0.003), platelets ≥350 × 10^9^/l (*P* = 0.027) and C-reactive protein (CRP) > 5 mg/L (*P* = 0.046) (Table 2A). The association with tumour type was not accounted for by recent surgery or adjuvant chemotherapy. As a sensitivity analysis, the 17 patients with ongoing chemotherapy at baseline were removed; effect sizes remained similar with tumour type remaining significant.

**Table 2:** Univariable and multivariable models for predicting U-TXM (based on log-transformed values). Panel A at registration and panel B at the end of the run-in period.

(A)						
Baseline characteristic	Univariable models			Multivariable model^d^		
	Estimate	(95% CI)	*P* value	Estimate	(95% CI)	*P* value
**Tumour type**						
Breast	Reference	–				
Colorectal	0.28	(0.16, 0.40)	<0.001	0.35	(0.22, 0.48)	<0.001
Gastro-oesophageal	0.73	(0.54, 0.92)	<0.001	0.71	(0.51, 0.92)	<0.001
Prostate	−0.01	(−0.13, 0.11)	0.856	0.08	(−0.12, 0.14)	0.904
**Gender**						
Male	Reference	–				
Female	−0.11	(−0.21, −0.02)	0.023			
**Age at registration (years)**						
<50 years	Reference	−				
50–59 years	0.03	(−0.12, 0.18)	0.656			
60–69 years	0.02	(−0.13, 0.16)	0.836			
70 years or over	0.15	(−0.01, 0.31)	0.063			
**BMI**						
BMI < 25	Reference	–				
BMI 25–29.9	−0.03	(−0.15, 0.09)	0.634			
BMI 30–34.9	−0.11	(−0.25, 0.04)	0.154			
BMI 35 or over	0.14	(−0.04, 0.32)	0.116	0.22	(0.06, 0.39)	0.008
**Smoking status**						
Never smoked	Reference	–				
Previous smoker	0.13	(0.03, 0.23)	0.011			
Current smoker	0.46	(0.24, 0.68)	<0.001			
**Diabetic**						
Not diabetic	Reference	–				
Diabetic	0.09	(−0.13, 0.31)	0.414			
**Receiving BP treatment**						
No BP treatment	Reference	–				
On BP treatment	−0.02	(−0.14, 0.09)	0.688			
**Statin use in last week**						
No recent statin use	Reference	–				
Statins within last week	−0.06	(−0.19, 0.08)	0.428			
**Neutrophils (10**^**9**^**/L)**						
Neutrophils <5.0 × 10^9^/L	Reference	–				
Neutrophils ≥5.0 × 10^9^/L	0.31	(0.15, 0.46)	<0.001	0.25	(0.09, 0.41)	0.003
**Total WCC (10**^**9**^**/L)**						
Total WCC < 8.0 × 10^9^/L	Reference	–				
Total WCC ≥ 8.0 × 10^9^/L	0.42	(0.24, 0.60)	<0.001			
**Platelet count (10**^**9**^**/L)**						
Platelet count <350 × 10^9^/L	Reference	–				
Platelet count ≥350 × 10^9^/L	0.32	(0.10, 0.54)	0.005	0.26	(0.03, 0.50)	0.027
**Raised LDL, total cholesterol or non-HDL**						
Normal cholesterol levels	Reference	–				
Raised LDL, total cholesterol or non-HDL^a^	−0.02	(−0.13, 0.08)	0.647			
**CRP result (categorised)**						
CRP ≤ 5	Reference	–				
CRP > 5	0.18	(0.03, 0.33)	0.017	0.16	(0.003, 0.31)	0.046
**Higher-risk disease**						
Lower-risk disease	Reference	–				
Higher-risk disease^b^	0.10	(0.00, 0.20)	0.049			
**Primary treatment details**						
Surgery, no adjuvant chemo	Reference	–				
Surgery and adjuvant chemo	0.21	(0.10, 0.32)	<0.001			
Radical RT or CRT	−0.05	(−0.20, 0.11)	0.538			
**Timing of surgery/primary treatment**						
6–12 weeks since surgery^c^	Reference	–				
>12 weeks since surgery	0.11	(−0.01, 0.24)	0.074			
Radical RT or CRT	−0.09	(−0.26, 0.08)	0.282			

(B)						
Baseline characteristic	Univariable models^d^			Multivariable model^d^		
	Estimate	(95% CI)	*P* value	Estimate	(95% CI)	*P* value
**Tumour type**						
Breast	Reference	–				
Colorectal	0.11	(−0.02, 0.24)	0.086			
Gastro-oesophageal	0.11	(−0.10, 0.32)	0.306			
Prostate	0.00	(−0.12, 0.12)	0.975			
**Gender**						
Male	Reference	–				
Female	−0.07	(−0.17, 0.03)	0.150			
**Age at registration (years)**						
<50 years	Reference	–				
50–59 years	0.03	(−0.12, 0.18)	0.663			
60–69 years	−0.01	(−0.15, 0.13)	0.915			
70 years or over	0.13	(−0.03, 0.28)	0.120	0.13	(0.003, 0.27)	0.045
**BMI**						
BMI < 25	Reference	–				
BMI 25–29.9	−0.09	(−0.21, 0.03)	0.162			
BMI 30–34.9	0.03	(−0.11, 0.18)	0.642			
BMI 35 or over	0.05	(−0.13, 0.22)	0.604			
**Smoking status**						
Never smoked	Reference	–				
Previous smoker	0.12	(0.02, 0.22)	0.024	0.16	(0.05, 0.26)	0.005
Current smoker	0.22	(−0.01, 0.44)	0.064	0.31	(0.04, 0.57)	0.025
**Diabetic**						
Not diabetic	Reference	–				
Diabetic	0.30	(0.08, 0.52)	0.008	0.31	(0.06, 0.56)	0.014
**Receiving BP treatment**						
No BP treatment	Reference	–				
On BP treatment	0.08	(−0.03, 0.20)	0.156			
**Statin use in last week**						
No recent statin use	Reference	–				
Statins within last week	0.11	(−0.03, 0.24)	0.134			
**Neutrophils (10**^**9**^**/L)**						
Neutrophils <5.0 × 10^9^/L	Reference	–				
Neutrophils ≥5.0 × 10^9^/L	0.21	(0.05, 0.37)	0.011	0.23	(0.06, 0.40)	0.008
**Total WCC (10**^**9**^**/L)**						
Total WCC < 8.0 × 10^9^/L	Reference	–				
Total WCC ≥ 8.0 × 10^9^/L	0.18	(−0.01, 0.37)	0.067			
**Platelet count (10**^**9**^**/L)**						
Platelet count <350 × 10^9^/L	Reference	–				
Platelet count ≥350 × 10^9^/L	−0.04	(−0.27, 0.19)	0.748			
**Raised LDL, total cholesterol or non-HDL**						
Normal cholesterol levels	Reference	–				
Raised LDL, total cholesterol or non-HDL^a^	−0.04	(−0.14, 0.07)	0.457			
**CRP result (categorised)**						
CRP ≤ 5	Reference	–				
CRP > 5	0.22	(0.06, 0.37)	0.006			
**Higher-risk disease**						
Lower-risk disease	Reference	–				
Higher-risk disease^b^	−0.09	(−0.19, 0.01)	0.083			
**Primary treatment details**						
Surgery, no adjuvant chemo	Reference	–				
Surgery and adjuvant chemo	0.02	(−0.09, 0.13)	0.664			
Radical RT or CRT	0.04	(−0.11, 0.20)	0.577			
**Timing of surgery/primary treatment**						
6–12 weeks since surgery^c^	Reference	–				
>12 weeks since surgery	0.06	(−0.07, 0.18)	0.380			
Radical RT or CRT	0.07	(−0.10, 0.24)	0.418			

Univariable and multivariable models for predicting U-TXM (based on log-transformed values of U-TXM) at baseline (trial registration) (Panel A) and at the end run-in period (Panel B).

^a^LDL > 3, total cholesterol >5 or non-HDL > 4; note that participants with at least one of LDL, HDL and total cholesterol measured and in the normal range (and no raised measurements) are included in the normal group even if other measurements are missing.

^b^Breast Stage III; colorectal Stage III or IV; gastro-oesophageal Stage IIB or III; prostate high risk according to D’Amico classification.

^c^Timing of surgery was not considered in the multivariate model due to being confounded with the primary treatment variable and since it was non-significant in the univariate model.

^d^The final multivariable model at registration is based on *n* = 552 observations (some observations are not included due to missing covariate information—see earlier table); *R*^2^ = 16.8%. At the end of the run-in period the final multivariable model is based on *n* = 402 observations *R*^2^ = 32.3%. All models are adjusted for (log-transformed) baseline U-TXM.

### The effect of low-dose aspirin (100 mg daily) on in vivo platelet activation

**Fig. 3:**
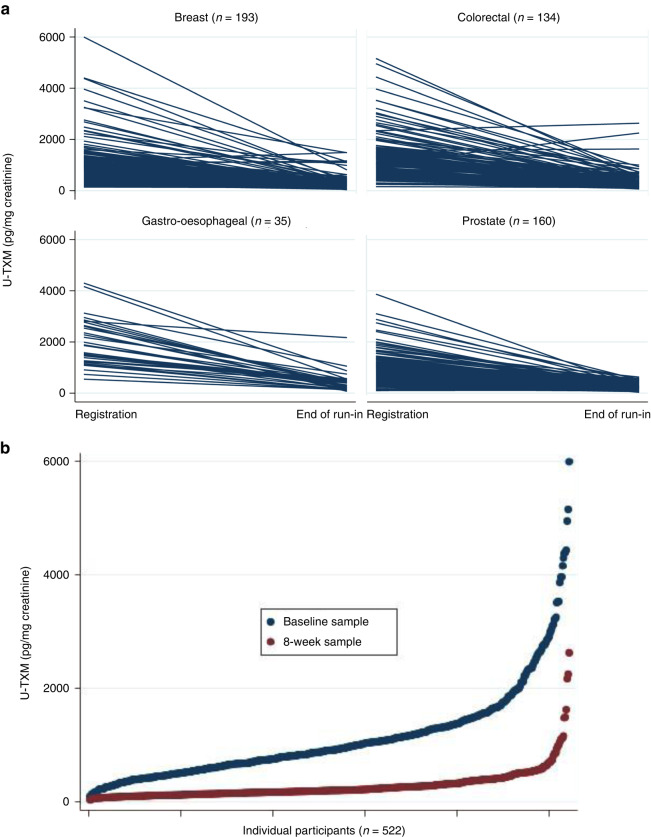
U-TXM levels at the end of the run-in period after 8 weeks of aspirin 100 mg daily. Panel **a** by tumour cohort and Panel **b** for the whole group.

At the end of the run-in period, urine samples from 522 participants were available (breast 193, colorectal 134, gastro-oesophageal 35, prostate 160) with 8 participants excluded due to reported poor adherence. Characteristics of participants who provided samples at the end of run-in were similar to the overall study cohort except that there were slightly fewer ex/current smokers (data not shown). Aspirin 100 mg daily suppressed U-TXM excretion similarly across all four cohorts (Fig. 3a, b) with a median percentage reduction of 76% consistent with previous data in other clinical settings [31]. Univariable and multivariable models were used to examine U-TXM at the end of the run-in period (Table 2B) with age ≥70 years (*P* = 0.045), current/previous smoking (*P* = 0.005 and *P* = 0.025, respectively), diabetes (*P* = 0.014) and neutrophils ≥5.0 × 10^9^/l (*P* = 0.008) being independently associated with higher levels in the multivariable analysis after adjustment for baseline U-TXM. For 14/522 (2.7%) of participants, U-TXM was the same or higher than baseline (6 breast, 4 colorectal and 4 prostate) at the end of run-in period. There was higher proportion of diabetics and current/previous smokers in this group compared to the whole cohort and it is also possible that adherence was not as reported.

### The effect of aspirin dose on in vivo platelet activation

**Fig. 4:**
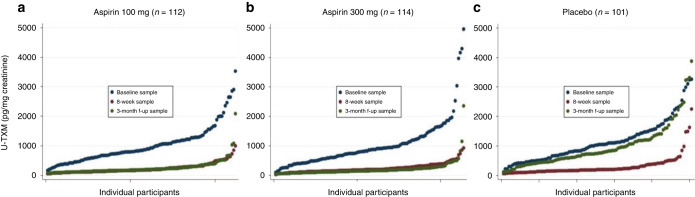
U-TXM levels over time at 3 time points (baseline, end of run-in and 3–6 months after random allocation). Panel **a**–**c** random allocation to aspirin 100 mg, 300 mg and matched placebo respectively.

Urine samples from 327 participants were available after at least 3 months post-random allocation (1:1:1) to 100 mg aspirin, 300 mg aspirin or matched placebo. Median U-TXM was similar in the two aspirin groups (194 and 159 pg/mg for 100 mg and 300 mg, respectively) and much lower than in the placebo group, where it was close to pre-treatment levels (median 752 pg/ml) (Supplementary Appendix S6). Absolute and percentage changes in U-TXM compared to the end of run-in sample were minimal on aspirin 100 and 300 mg daily while in the placebo arm there was an approximately threefold increase (median absolute increase of 530 pg/mg) as expected. A summary of changes in U-TXM over time for each of the three groups (placebo, aspirin 100 mg and aspirin 300 mg) is shown in Fig. 4. It illustrates similar levels of suppression of platelet activation for the two aspirin doses and a return to near baseline levels when participants received placebo and provides reassurance that the majority of participants are complying with the treatment schedule and not consuming additional over-the-counter aspirin. Absolute and percentage change between the baseline and the 3-month follow-up timepoint are shown in Supplementary Appendix S7. An analysis of the effects of aspirin dose (100 mg versus 300 mg daily) considered by baseline factors known to affect platelet activation (Supplementary Appendix S8) shows slightly lower median values for the 300 mg aspirin group, though no definitive evidence that a higher dose would be more effective in some subgroups (noting that numbers in some of the categories are small).

## Discussion

To our knowledge, this is the largest and most detailed study of thromboxane biosynthesis in patients with early-stage cancer. Increased levels of U-TXM excretion were found in all the tumour cohorts compared to previous studies of healthy individuals measured in the same laboratory [31, 41, 42]. The levels were higher than expected given all of the patients had been treated with radical intent and had no clinical evidence of metastatic disease when they entered the trial. Expected 5-year disease-free survival rates vary by tumour type but range from around 55% for the gastro-oesophageal participants to over 80% for those with breast and prostate cancer. With the exception of 17 (2%) of the participants, cancer treatment (chemotherapy or radiotherapy) had finished several weeks (no more than 6–14 weeks) before entry into the study, and all patients were at least 6 weeks from surgery with over 50% more than 12 weeks. The data also show that for the patients allocated to placebo after the run-in period U-TXM levels return to near baseline levels suggesting persistently enhanced thromboxane biosynthesis. The highest levels were associated with tumours that arose from the gastrointestinal tract (i.e., the colorectal and gastro-oesophageal cohorts) compared to the breast and prostate cohorts. In the multivariable analysis, this was not accounted for by baseline participant characteristics or treatment factors such as time from recent radical therapy (surgery or radiotherapy) or receiving adjuvant chemotherapy.

Consistent with previous literature, high U-TXM levels at baseline were also associated with known inflammatory stimuli, or markers thereof, including high BMI, a history of smoking, raised total white cells, neutrophils, and higher platelet counts [41, 43–46]. In the multivariable analysis, raised inflammatory markers, higher platelet numbers and BMI >35 remained statistically significant. The variation of thromboxane biosynthesis across the tumour cohorts is an interesting observation given studies suggest that aspirin may be most effective in preventing cancers that arise from the gastrointestinal tract [17, 18, 20].

The biological plausibility of, and supporting evidence, for the role of platelet activation in colorectal cancer has been reviewed previously [1, 47, 48]. The differential increase of U-TXM observed in colorectal and gastro-oesophageal cancers vis-à-vis breast and prostate is consistent with the hypothesised role of platelets in intestinal tumorigenesis [1]. The role of platelets in breast and prostate cancer has been less intensively investigated, but a recent breast cancer study that correlated U-TXM, urinary markers of oxidative stress and clinical characteristics suggested enhanced oxidative stress due to insulin resistance and oestrogen-related mechanisms may cause platelet activation and a positive environment for tumour growth [49]. Similarly in a prostate cancer case–control study higher levels of U-TXM were associated with higher-risk disease and poorer outcomes [50]. Studies also show increased levels of TXA_2_ synthase and TXA_2_ receptors in multiple tumour types (though rarely mutated) leading to the hypothesis that thromboxane receptor antagonists which would also decrease TXA_2_ production in tumours should also be assessed for their anti-metastatic potential [15].

Aspirin’s main mechanism of action at low dose (75–100 mg daily) is the acetylation of platelet COX-1 at serine-529 [9] resulting in 70–80% suppression of U-TXM, with recovery of excretion corresponding to the platelet lifespan (7–10 days) [8, 51]. Under pathophysiological conditions COX-2 in renal and inflammatory cells may contribute to enhanced TXA_2_ production [52], however, both are largely unaffected by low aspirin doses [46, 52]. Although urinary prostanoid metabolites do not reflect a specific cellular source, there is considerable evidence that urinary TXA_2_/TXB_2_ metabolites largely originate from platelet-derived TXA_2_ as reviewed previously [31]. Recent data to support this hypothesis derives from the pPtgs1^−/−^ mice with the deletion of COX-1 in megakaryocytes and platelets. These mice have a selective reduction of platelet TXA_2_ biosynthesis, as reflected by a 70% reduction in U-TXM excretion, but the systemic biosynthesis of PGE_2_, PGI_2_ and PGD_2_, as assessed by measuring their major urinary enzymatic metabolites, i.e., PGEM, PGIM and PGDM, respectively, is not significantly affected [14]. Our finding that the extent of U-TXM suppression (averaging 76%) by aspirin 100 mg was not further increased by a threefold higher daily dose is consistent with the saturability of platelet COX-1 inactivation at the lower dose. Although low-dose aspirin (100 mg daily) can acetylate COX-1 in the colorectal mucosa [53], the level of acetylation is lower than in circulating platelets and is associated with marginal (i.e., <10%) reduction in urinary PGEM.

The present findings in cancer patients are consistent with the saturability of platelet COX-1 inactivation at low aspirin doses with a three-quarter reduction in TXA_2_ biosynthesis with 100 mg aspirin daily and no further meaningful reduction observed with 300 mg daily [51, 54]. In addition, our findings are similar to those in healthy volunteers where aspirin 100 mg daily reduced U-TXM excretion by 65–70% following 1- to 8-week dosing [55, 56], suggesting a similar primary source of increased TXA_2_ biosynthesis.

Increasing age, cigarette smoking and obesity all lead to chronic, low-grade inflammation, and are independently well-defined aetiological factors associated with malignancy, attributed in part to their effects on platelet activation [57]. Our data could potentially help to identify individuals most likely to benefit from aspirin for the primary prevention of cancer, i.e., those with high levels of thromboxane production associated with known risk factors for cancer. Our observation that higher platelet counts were associated with increased levels of thromboxane biosynthesis aligns with recent data suggesting that platelet counts (or possibly platelet characteristics) could be used as an early indicator/screening tool to detect cancer [58, 59]. The 1-year incidence of cancer in patients with thrombocytosis (>400 × 10^9^/L) has been shown to exceed the UK national threshold for investigating possible cancer cases with data also suggesting that in older males (≥60 years) a platelet count in the high-normal range (>325 × 10^9^/L) is also associated with an increased cancer risk [59, 60]. The utility of platelet counts and markers of activation to monitor cancer progression is more challenging as many cancer-related treatments effect platelets [61].

The mechanism(s) by which aspirin exerts an anti-cancer effect remains speculative, but our data suggest a plausible link between the known pharmacological effects of aspirin and previous clinical observations by demonstrating persistently enhanced thromboxane A_2_ generation in patients recently diagnosed with cancer. The data also support the ongoing evaluation of two aspirin dose levels within the Add-Aspirin trial for two reasons. Firstly, our results demonstrate an adequate pharmacodynamic response to low-dose aspirin in cancer patients, comparable to that achieved in other clinical settings, such as cardiovascular disease where clinical efficacy has been demonstrated. Secondly, the lack of a further reduction in TXA_2_ biosynthesis in response to the higher aspirin dose, means that any dose-related pattern in efficacy and/or safety can be interpreted as reflecting an extra-platelet target of aspirin. Several mechanisms independent of inhibition of platelet activation have been hypothesised to explain the anti-cancer effects of aspirin [62, 63] and targeting these pathways may then require a higher dose of aspirin.

Since this study was embedded within a randomised-controlled trial which incorporates a placebo arm, it provides a robust and valuable dataset for investigating the effects of aspirin on thromboxane biosynthesis. Nevertheless, there are some limitations. U-TXM is one major enzymatic metabolite of TXA_2_/TXB_2_ that can also be metabolised to 2,3-dinor-TXB_2_. Our study did not include measurements of 2,3-dinor-TXB_2_, but our previous work in diabetic patients with enhanced excretion of U-TXM found a linear conversion of exogenously infused TXB_2_ to U-TXM over a 50-fold range of infusion rates (with a fractional elimination similar to healthy subjects) suggesting increased biosynthesis of TXA_2_/TXB_2_ rather than a shift in its metabolic disposition [46]. The current finding of raised U-TXM associated with high BMI and inflammatory markers in cancer patients is similar to other non-cancer clinical settings, e.g., obesity and type II diabetes mellitus studied previously [43, 45, 46] and leads us to believe that the fractional conversion of endogenous TXA_2_/TXB_2_ to U-TXM would be similarly unaltered in cancer patients.

Other limitations of this study are in part related to the design of the main trial including the range of aspirin doses that could be evaluated. Additionally, measurement of serum TXB_2_ or markers of platelet aggregation would have required on-site pre-analytical processing [64] and was not logistically possible in this large pragmatic trial. However, given that U-TXM excretion was suppressed by aspirin 100 mg daily to the same extent as previously reported in healthy subjects, it is reasonable to assume that platelet COX-1 activity was comparably suppressed. By necessity, the analyses of samples from beyond the baseline timepoint could only include participants who continued with trial treatment. This might impact the generalisability of the results, but the numbers stopping treatment or participation were small and we did not identify any important differences between these participants compared to the whole trial cohort. Finally, the lack of longer-term clinical outcomes, i.e., recurrence and survival data, for correlation with U-TXM levels at the present time is not possible as the randomised trial is still ongoing.

In summary, this study found that patients who have had a recent diagnosis of cancer and radical therapy showed evidence of persistently increased thromboxane biosynthesis as measured by U-TXM excretion compared to healthy individuals. This was most marked in the colorectal and gastro-oesophageal participants independent of pro-inflammatory baseline characteristics and recent anti-cancer treatment. Our findings show comparable suppression of U-TXM with aspirin 100 and 300 mg daily and are consistent with the saturability of platelet COX-1 inactivation at low doses. Within this cohort of participants adherence with randomly allocated blinded treatment appeared good. These findings may help interpret the clinical results of the Add-Aspirin trial and other aspirin oncology trials subsequently. This is particularly important given the differences we have observed between the tumour types in this study; as with other anti-cancer drugs the effects of aspirin may vary with different cancer types, molecular pathways driving the disease and the specific tumour microenvironment. Further studies are required to assess whether regular monitoring of U-TXM levels might provide an early indicator of tumour development or recurrence and a novel biomarker particularly in gastrointestinal cancers.

## Disclaimer

The views expressed are those of the authors and not necessarily those of the NHS, the NIHR or the Department of Health and Social Care.

## Supplementary information

Supplementary Appendix

## Data Availability

The trial data are held at MRC CTU at UCL, which encourages optimal use of data by use of a controlled access approach to data sharing. Requests for data can be made at any time and can be initiated by contacting mrcctu.ctuenquiries@ucl.ac.uk or via the MRC CTU website. There is a formal application process, whereby the request will undergo review by the trial team, as well as independent researchers, to ensure that the proposed research is both ethical and has a strong scientific rationale. Data will not be released if it would compromise the ongoing trial. The specific data and associated documents to be shared will be dependent on the nature of the individual request and this will be documented in a formal data-sharing agreement.
