# Supplementary material for: Thromboxane biosynthesis in cancer patients and its inhibition by aspirin: a sub-study of the Add-Aspirin trial
Source: Br J Cancer. 2023 Jul 7;129(4):706–20. doi: 10.1038/s41416-023-02310-1 (PMC10421951; doi:10.1038/s41416-023-02310-1)
Supplement: Supplementary file 1 — Supplementary Appendix [file 41416_2023_2310_MOESM1_ESM.docx]

**Supplementary Appendix**

**S1. Additional Details of the U-TXM extraction and assay**

*Urine extraction*

Urine samples of 1 mL were thawed at 37°C and centrifuged at 340*g* for 10 minutes; 60 ul of acetic acid was added to reach a pH of approximately 3.5, 1,000 cpm ^3^H-TXB_2_ were added to 1 mL urine sample. Samples were loaded into a 1 mL/50 mg C18 column, prewashed with 1 mL absolute methanol and 2.5 mL distilled H_2_O. Column was then washed with 1.6 mL distilled H_2_O and 2 mL acetonitrile/water (15:85, vol/vol), dried and eluted with 2.5 mL isooctane/ethyl acetate (1:1, vol/vol). The eluate was loaded on a 1 mL/100 mg SiOH column prewashed with 2 mL isooctane/ethyl acetate (1:1,vol/vol), eluted with 2 mL ethylacetate/methanol (60:40, vol/vol). After drying, eluate was resuspended in 1 mL PBS/0.1% BSA buffer, 500ul of the final resuspension were counted for calculating the recovery. The ELISA measurements were corrected for the % of recovery based on ^3^H-TXB_2_ cpm counts. This method has been published before (1).

To assess the stability and reproducibility of the extraction procedure over the entire sub-study duration, a pool of urine from healthy donors was aliquoted into 1mL samples and frozen until use. In each experimental set, aliquots were thawed and spiked with vehicle (ethanol 0.05% vol/vol) or with known concentrations of the exogenous cold TXM commercial standard at final concentrations ranging from 0.5 and 2 ng/ml. These experiments were performed at least every two months over the entire duration of the analyses.

*TXM measurements*

Urinary TXM was measured in the extracted samples by a standard Enzyme Linked Immunosorbent Assay (ELISA) assays (2, 3). Ninety-six-well plates were coated with commercial monoclonal anti-rabbit IgG antibodies according to the standard method for coating. TXM in urinary extracts was measured with a standard AchE ELISA immunometric method, using a specific rabbit polyclonal antibod4. The ELISA assay using this antibody had a range of detection from 0.5 to 0.0039 ng/ml, a sensitivity calculated as B/B_0_ (Bound/Maximum Bound) 80% of 0.01 ng/ml, and an overall inter-assay coefficient of variation of 8.8%. Samples that measured <0.018 ng/ml were assayed with a different commercial anti-rabbit IgG, which had a lower range of detection from 0.25 to 0.0019 ng/ml and a 80% B/B0 of 0.004 ng/ml. The cross-reactivity of the anti-11-dehydro TXB_2_ antibodies against other prostanoids that can be measured in urines, namely PGE_2_ 2,3-dinor TXB2, Thrombxane B2, 6-keto PGF1alpha, and the isoprostane 8-iso-PGF_2alpha_ was <0.05%, cross-reactivity with PGD2 was 0.3%. Moreover, the results obtained by the EIA method using the polyclonal antibody were already shown to be highly correlated with gas chromatography/mass spectrometry analysis (4).

The validation of the method was based on the U.S. Food and Drug Administration guidelines for Validation of Bioanalytical Methods (5). Internal standards were used for intra-assay validation and consisted of pools of urinary extracts from healthy donors which were aliquoted, frozen and one aliquot was used per each plate.

TXM final values were corrected for the concentration of urinary creatinine, that was measured by a commercial kit based on the Jaffe’s reaction (6). Two internal standards were used in each assay plate: a pool of urine and a commercial standard. These internal standards were always included to assess the consistency and reproducibility of the assays over time.

**References**

1. Pagliaccia F, Habib A, Pitocco D, Petrucci G, Zaccardi F, Di Stasio E, et al. Stability of urinary thromboxane A2 metabolites and adaptation of the extraction method to small urine volume. Clin Lab. 2014;60(1):105-11

2. Pradelles P, Grassi J, Maclouf J. Enzyme immunoassays of eicosanoids using acetylcholine esterase as label: an alternative to radioimmunoassay. Anal Chem. 1985;57(7):1170-3.

3. Lellouche F, Fradin A, Fitzgerald G, Maclouf J. Enzyme immunoassay measurement of the urinary metabolites of thromboxane A2 and prostacyclin. Prostaglandins. 1990;40(3):297-310. .

4. Wang Z, Ciabattoni G, Creminon C, Lawson J, Fitzgerald GA, Patrono C, et al. Immunological characterization of urinary 8-epi-prostaglandin F2 alpha excretion in man. J Pharmacol Exp Ther. 1995;275(1):94-100.

5. U.D.o.H.a.H.S. Food and Drug Administration CfDEaR. Guidance for Industry: Bioanalytical Method Validation. 2013.

6. Cook JG, Association of clinical Biochemists S, Technica C. Factors influencing the assay of creatinine. Ann Clin Biochem. 1975;12(6):219-32.

**S2: Consort Diagram**

**Participants included in the thromboxane biosynthesis study**

**S3:**

**Urinary TXM excretion rates in healthy individuals from previous studies off aspirin**

| **Reference** | **Number of subjects**  **(M/F)** | **Age**  **Mean± SD**  **(range)**  **years** | **11-dehydro-TXB_2_**  **pg/mg creatinine**  **Mean ± SD or Median (IQR)** | **Method** |
| --- | --- | --- | --- | --- |
| **Ref 1-4 and unpublished data** | 65  (47M, 18F) | 39 ± 13 | 482 ± 235 | EIA polyclonal |
| **Ciabattoni et al. 2007 [5]** | 44  (27F, 17M) | 75 ± 7 | 517.5  [402.5-653] | RIA- polyclonal |
| **Rocca et al. 1995 [6]** | 26 | 53 ±15 | 504 ± 267 | RIA-polyclonal |

M=male F=female EIA = immunoenzyne assay RIA = radioimmune assay

**References:**

1. Santilli, F., G. Davì, S. Basili, S. Lattanzio, A. Cavoni, G. Guizzardi, L. De Feudis, G. Traisci, C. Pettinella, L. Paloscia, P. Minuz, A. Meneguzzi, G. Ciabattoni and C. Patrono (2010). "Thromboxane and prostacyclin biosynthesis in heart failure of ischemic origin: effects of disease severity and aspirin treatment." J Thromb Haemost **8**(5): 914-922.
2. Santilli, F., B. Rocca, R. De Cristofaro, S. Lattanzio, L. Pietrangelo, A. Habib, C. Pettinella, A. Recchiuti, E. Ferrante, G. Ciabattoni, G. Davì and C. Patrono (2009). "Platelet cyclooxygenase inhibition by low-dose aspirin is not reflected consistently by platelet function assays: implications for aspirin "resistance"." J Am Coll Cardiol **53**(8): 667-677.
3. Zaccardi F, Rizzi A, Petrucci G, Ciaffardini F, Tanese L, Pagliaccia F, Cavalca V, Ciminello A, Habib A, Squellerio I, Rizzo P, Tremoli E, Rocca B, Pitocco D, Patrono C. [In Vivo Platelet Activation and Aspirin Responsiveness in Type 1 Diabetes.](https://pubmed.ncbi.nlm.nih.gov/26470782/) Diabetes. 2016 Feb;65(2):503-9.
4. Simeoni S, Biselli R, D'Amelio R, Rocca B, Lattanzio S, Mucci L, Davì G, Patacchioli FR. Stress-induced salivary cortisol secretion during hypobaric hypoxia challenge and in vivo urinary thromboxane production in healthy male subjects. Stress. 2011 May;14(3):282-9.
5. Ciabattoni, G., E. Porreca, C. Di Febbo, A. Di Iorio, R. Paganelli, T. Bucciarelli, L. Pescara, L. Del Re, C. Giusti, A. Falco, A. Sau, C. Patrono and G. Davì (2007). "Determinants of platelet activation in Alzheimer's disease." Neurobiology of Aging **28**(3): 336-342.
6. Rocca, B., G. Ciabattoni, R. Tartaglione, S. Cortelazzo, T. Barbui, C. Patrono and R. Landolfi (1995). "Increased thromboxane biosynthesis in essential thrombocythemia." Thromb Haemost **74**(5): 1225-1230.

**S4. Additional detail for Figure 2**


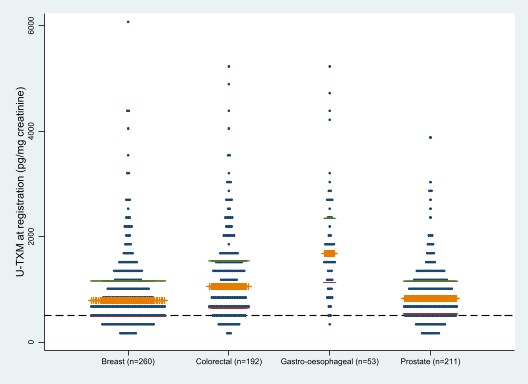


**Distribution of U-TXM at registration by tumour-specific cohort**

Yellow points represent the median value for each cohort; red and green lines represent the lower and upper quartiles respectively; the black dotted line represents the median value in healthy individuals as indicated in section S3 above and reference 31 of the main text.


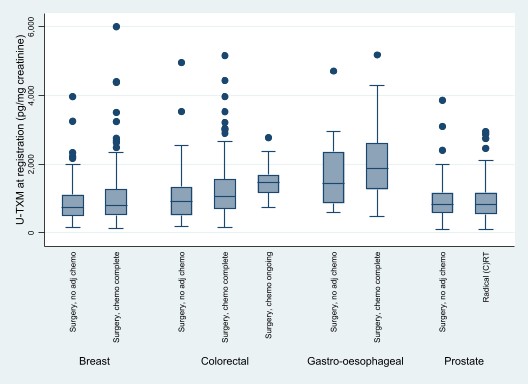


**S5. Distribution of U-TXM at registration according to primary treatment detail**

Categories not displayed if numbers were too small to derive box and whisker plots.

**S6. Change in U-TXM after 3 months of randomised treatment (compared with end of run-in values)**

| **Randomised treatment** | **n** | **Median** | **(Q1, Q3)** | **[Range]** |
| --- | --- | --- | --- | --- |
| **U-TXM after 3 months of randomised treatment** | | | | |
| 100mg aspirin | 112 | 194 | (145, 282) | [77, 2090] |
| 300mg aspirin | 114 | 159 | (112, 230) | [47, 2358] |
| Placebo | 101 | 752 | (475, 1201) | [125, 3876] |
| **U-TXM absolute changes after 3 months randomised treatment (vs end run-in)*** | | | | |
| 100mg aspirin | 112 | -1 | (-59, 52) | [-387, 1977] |
| 300mg aspirin | 114 | -29 | (-109, 29) | [-748, 1654] |
| Placebo | 101 | 530 | (279, 894) | [-1831, 3713] |
| **U-TXM percentage changes after 3 months randomised treatment (vs end run-in)*** | | | | |
| 100mg aspirin | 112 | 0% | (-29%, 30%) | [-74%, 1754%] |
| 300mg aspirin | 114 | -21% | (-42%, 17%) | [-80%, 272%] |
| Placebo | 101 | 288% | (136%, 445%) | [-81%, 2273%] |

* Negative values indicate a reduction since end of run-in sample

Negative values indicate a decrease during this period

**S7. Change in U-TXM after 3 months of randomised treatment (compared with baseline values)**

| **Randomised treatment** | **n** | **Median** | **(IQR)** | **[Range]** |
| --- | --- | --- | --- | --- |
| 100mg aspirin | 112 | -647 | (-977, -407) | [-3004, 1698] |
| 300mg aspirin | 114 | -649 | (-990, -397) | [-4032, 33] |
| Placebo | 101 | -123 | (-370, 119) | [-2547, 2399] |

Negative values indicate a decrease during this period

**Percentage change** **in U-TXM after 3 months of randomised treatment (compared with baseline values)**

| **Randomised treatment** | **n** | **Median** | **(IQR)** | **[Range]** |
| --- | --- | --- | --- | --- |
| 100mg aspirin | 112 | -76% | (-85%, -65%) | [-95%, 434%] |
| 300mg aspirin | 114 | -80% | (-86%, -69%) | [-95%, 14%] |
| Placebo | 101 | -12% | (-43%, 14%) | [-88%, 485%] |

Negative values indicate a decrease during this period

**S8. Median U-TXM after 3 months of randomised treatment according to dose and baseline factors**

| **Baseline characteristic** | **100mg aspirin** | | | | **300mg aspirin** | | | |
| --- | --- | --- | --- | --- | --- | --- | --- | --- |
|  | **n** | **Median** | **(IQR)** | **[Range]** | **n** | **Median** | **(IQR)** | **[Range]** |
|  |  |  |  |  |  |  |  |  |
| Under 50 yrs | 28 | 231 | (167, 324) | [77, 672] | 20 | 159 | (116, 203) | [69, 1162] |
| 50-59 yrs | 38 | 185 | (139, 222) | [84, 1081] | 27 | 152 | (108, 235) | [53, 447] |
| 60-69 yrs | 36 | 169 | (131, 235) | [101, 2090] | 47 | 157 | (112, 205) | [47, 2358] |
| 70-74 yrs | 10 | 298 | (236, 529) | [116, 1038] | 20 | 193 | (131, 253) | [61, 483] |
|  |  |  |  |  |  |  |  |  |
| BMI <25 | 30 | 175 | (125, 279) | [97, 2090] | 34 | 149 | (106, 195) | [47, 2358] |
| BMI 25-29.9 | 35 | 186 | (149, 236) | [84, 1081] | 44 | 156 | (112, 206) | [53, 1162] |
| BMI 30-34.9 | 25 | 194 | (159, 267) | [77, 522] | 18 | 169 | (120, 230) | [74, 505] |
| BMI >35 | 16 | 297 | (153, 385) | [78, 672] | 8 | 216 | (185, 261) | [169, 483] |
|  |  |  |  |  |  |  |  |  |
| Never | 64 | 187 | (140, 239) | [77, 1081] | 60 | 156 | (108, 190) | [47, 827] |
| Ex-smoker | 41 | 208 | (146, 340) | [78, 2090] | 46 | 173 | (118, 254) | [68, 1162] |
| Current smoker | 7 | 196 | (164, 388) | [130, 450] | 8 | 241 | (184, 370) | [108, 2358] |
|  |  |  |  |  |  |  |  |  |
| Not diabetic | 104 | 191 | (143, 289) | [77, 2090] | 102 | 159 | (114, 226) | [53, 1162] |
| Diabetic | 3 | 241 | (222, 512) | [222, 512] | 5 | 157 | (129, 187) | [47, 313] |
|  |  |  |  |  |  |  |  |  |
| Breast | 44 | 181 | (150, 263) | [78, 2090] | 42 | 145 | (106, 181) | [60, 573] |
| Colorectal | 29 | 219 | (117, 390) | [77, 1081] | 28 | 183 | (142, 253) | [53, 2358] |
| Gastro-oesophageal | 4 | 281 | (162, 418) | [103, 497] | 9 | 226 | (106, 311) | [74, 515] |
| Prostate | 35 | 194 | (146, 241) | [101, 512] | 35 | 165 | (120, 208) | [47, 827] |
|  |  |  |  |  |  |  |  |  |
